# Administration of *Lactobacillus fermentum* KBL375 Causes Taxonomic and Functional Changes in Gut Microbiota Leading to Improvement of Atopic Dermatitis

**DOI:** 10.3389/fmolb.2019.00092

**Published:** 2019-09-27

**Authors:** Woon-Ki Kim, You Jin Jang, Dae Hee Han, Boram Seo, SungJun Park, Chang Hyung Lee, GwangPyo Ko

**Affiliations:** ^1^Graduate School of Public Health, Seoul National University, Seoul, South Korea; ^2^N-Bio, Seoul National University, Seoul, South Korea; ^3^KoBioLabs, Inc., Seoul, South Korea; ^4^Department of Agricultural Biotechnology, Seoul National University, Seoul, South Korea; ^5^Center for Human and Environmental Microbiome, Seoul National University, Seoul, South Korea; ^6^Institute of Health and Environment, Seoul National University, Seoul, South Korea

**Keywords:** atopic dermatitis, *Lactobacillus fermentum*, immunomodulation, microbiota, metabolome, microbiome

## Abstract

Gut microbiota play an important role in immune responses and energy metabolism. In this study, we evaluated whether administration of *Lactobacillus fermentum* (*L. fermentum*) KBL375 isolated from healthy Korean feces improves the atopic dermatitis using the house dust mite (*Dermatophagoides farinae*)-induced atopic dermatitis (AD) mouse model. Administration of *L. fermentum* KBL375 significantly decreased dermatitis score, ear and dorsal thickness, and serum immunoglobulin E level in AD-induced mice. Significant reductions in mast cells and eosinophils were discovered in skin tissues from *L. fermentum* KBL375-treated mice. T helper 2 cell-related cytokines interleukin (IL)-4, IL-5, IL-13, and IL-31 significantly decreased, and anti-inflammatory cytokine IL-10 or transforming growth factor-β increased in skin tissues from *L. fermentum* KBL375-treated mice. In addition to phenotypic changes in skin tissues, *L. fermentum* KBL375 treatment induced an increase in the CD4+CD25+Foxp3+ cell population in mesenteric lymph nodes. Taxonomic and functional analyses of gut microbiota showed significantly higher cecum bacterial diversities and abundances including genus *Bilophila, Dorea*, and *Dehalobacterium* in *L. fermentum* KBL375-treated mice. Metabolic analysis of the cecum also showed significant changes in the levels of various amino acids including methionine, phenylalanine, serine, and tyrosine, as well as short chain fatty acids such as acetate, butyrate, and propionate in AD-induced mice due to *L. fermentum* KBL375 treatment. These altered metabolites in AD-induced mice returned to the levels similar to those in control mice when treated with *L. fermentum* KBL375. Therefore, *L. fermentum* KBL375 could be useful for AD treatment by modulating the immune system and inducing various metabolites.

## Introduction

Clinically, atopic dermatitis (AD) is characterized by chronic and abnormal inflammation of the skin with compromised skin barrier integrity, high inflammatory responses toward stimulants, and a reduction in antimicrobial responses (Rather et al., 2016). The incidence rate of AD has increased globally (Huang et al., 2017), but an effective treatment for AD has not yet been suggested. During the acute phase of AD, an increase in T helper (Th) 2 cells occurs in skin lesions (Meagher et al., 2002; Peng and Novak, 2015). The level of IL-4, the major Th2-related cytokine in inflammatory processes due to AD, is increased, and subsequently, there is an increase in IL-5 levels, which can induce B cells and Immunoglubulin (Ig) E synthesis (Kishimoto and Hirano, 1988). Moreover, the over-expression of thymic stromal lymphopoietin (TSLP), an IL-7-like cytokine, plays an important role in the activation of dendritic cell (DC)-mediated Th2 inflammatory responses, which can occur in keratinocytes (Liu, 2006). The concentrations of Th2-related chemokines, including macrophage-derived chemokine (MDC) and thymus and activation regulated chemokine (TARC), associated with the infiltration of inflammatory cells into skin lesions, also show a strong correlation with the severity of AD (Nakazato et al., 2008; Kim et al., 2013). Th1 and Th17 cells gradually increase during the chronic development of AD (Meagher et al., 2002; Peng and Novak, 2015), and Foxp3+ T regulatory cell (Treg) populations can inhibit AD development via production of anti-inflammatory cytokine IL-10 and transforming growth factor (TGF)-β (Zhang et al., 2014).

Gut microbiota can convey various beneficial effects for inflammatory diseases via modulating the host immune system (Sommer and Bäckhed, 2013; Zeng et al., 2017). To understand the effects of gut microbiota for the host, various animal models such as mouse were used. Mouse has significant similarities with human in their chromosomal genes and gut microbiota (Yue et al., 2014; Hugenholtz and de Vos, 2018). To evaluate the intervention methods for various diseases, abundant laboratory mouse models, such as wild-type and knock-out mice, are available (Perlman, 2016). Therefore, mouse models have been widely used for biological researches even though the clear differences in two species exist. Probiotics, defined as live microorganisms conferring health benefits to the host, can restore function of the gut microbiome and stimulate the production of metabolites such as short-chain fatty acids (SCFAs) (Hemarajata and Versalovic, 2013; Hwang, 2016). SCFAs are mainly generated by anaerobic bacteria in the host's intestine and can play important roles in adjusting host metabolism and regulating of the immune response (Vonk et al., 2011). Amino acids (AAs) are the major components of proteins and are involved in energy regulation in organisms, and are also used for SCFA production (Dai, 2011; Neis et al., 2015). Previous studies have reported that *Lactobacillus spp*., major probiotics known for their lactic acid production, have strong effects on the host's immune responses such as decreases in Th1, Th2, and Th17-related cytokines or increases in IL-10 or CD4+CD25+ regulatory T cells (Park et al., 2008; Lim et al., 2017; Kwon et al., 2018; Jang et al., 2019; Kim et al., 2019). Moreover, *Lactobacillus spp*. can alleviate AD via modulation of gut microbiota (Kwon et al., 2018), and affecting host metabolic pathways (Jang et al., 2019; Kim et al., 2019).

Therefore, we evaluated the effects of *Lactobacillus fermentum* (*L. fermentum*) KBL375, isolated from the feces of healthy donors (Jang et al., 2019), for AD treatment using an *in vivo* house dust mite (*Dermatophagoides farinae*, DFA) extract-induced AD mouse model using NC/Nga mice. After the treatment of *L. fermentum* KBL375, we assessed the ameliorations of AD symptoms, and immunomodulation effects such as the expression of cytokines or chemokines, and changes in gut microbiota in AD-induced mice for further applications to humans.

## Materials and Methods

### Preparation of *L. fermentum* KBL375

*L. fermentum* KBL375, isolated from healthy Korean feces, was cultured as previously described with minor modifications (Jang et al., 2019; Kim et al., 2019). Briefly, *L. fermentum* KBL375 was cultivated in Lactobacilli MRS Agar (BD Difco, Sparks, MD, USA) supplemented with 0.05% L-cysteine-hydrochloride at 37°C for 24 h under anaerobic conditions using the Anaeropack (Mitsubishi Gas Chemical Company, Inc., Tokyo, Japan). The concentration of *L. fermentum* KBL375 was measured using the cultivation method (Unit: Colony forming unit; CFU) and the optical density (OD)-based method at 600 nm. After cultivation, bacterial cells were harvested using centrifugation (1,200 × g) and washed twice with 1 × phosphate-buffered saline (PBS) prior to administration to mice.

### AD-Induced *in vivo* Mouse Model

All experimental procedures for the AD-induced *in vivo* mouse model were approved by the Institutional Animal Care and Use Committee (IACUC: SNU-160928-1-1) of Seoul National University, Republic of Korea. The AD-induced *in vivo* mouse model was performed as previously described with minor modifications (Kang et al., 2015). Briefly, 5 week-old male NC/Nga mice (Central Lab Animals Inc., Seoul, Republic of Korea), bred from one production colony, were prepared and grouped with nine mice, per experimental condition. Three mice shared a cage. For induction of AD in skin, we removed the ear and dorsal skin hair using electric clipper and hair removal cream and applied 150 μL of 4% sodium dodecyl sulfate (SDS) in the ear and dorsal skin for 3 h to disrupt skin barrier. Then, we administered 100 mg of *Dermatophagoides farinae* extract (DFE) cream (Biostir® AD; Biostir, Inc, Hiroshima, Japan) twice a week for 21 days. Next, we administered approximately 1 × 10^9^ CFUs of *L. fermentum* KBL375 in 200 μL of PBS to mice via oral gavage daily for 28 days with weekly application of DFE cream. After treatment with *L. fermentum* KBL375, mice were sacrificed and cecum, mesenteric lymph nodes (MLNs), serum, and skin were collected for further analyses.

### Measurement of Clinical Symptoms

To assess the severity of AD, we anesthetized mice using 2% isoflurane. We then obtained images of the ear or dorsal skin and measured skin thickness (Figure S1A). Dermatitis scores of mice were measured once a week with blinded scoring of four researchers using the following criteria: (1) erythema/hemorrhage, (2) scaling/dryness, (3) edema, and (4) excoriation/erosion (Table S1) (Kang et al., 2015). Total dermatitis scores for mice were calculated with the maximal score of 12. Prior to sacrifice, transepidermal water loss (TEWL), representing the volume of water emitted from the body to the atmosphere through the epidermal layer (skin) by diffusion and evaporation, was measured using a Tewameter TM300 (Courage and Khazaka, Cologene, Germany) at 21–22°C and 50–55% humidity as described previously (Jimbo et al., 2015). The probe was located on the skin for 30 s and the data were measured. Scratching behaviors of mice were also observed for 10 min as described previously with minor modifications (Andoh et al., 2011). Briefly, for acclimation, mice were placed separately in an acrylic case for 1 h. Their behaviors were then recoded individually for 30 min. Total scratching behaviors to the nose, ear, and dorsal skin were counted for 10 min. Repeated and rapid scratching movements over 1 s were counted as one scratching. Blood samples were collected from blood vessels in the eyes of mice. Then, the serum was separated from blood samples by centrifugation at 1,200 × g for 15 min at 4°C. Serum IgE levels were measured using the IgE enzyme-linked immunosorbent assay (ELISA) kit (Komabiotech, Seoul, Korea) according to the manufacturer's instructions.

### Histological Analysis

Dorsal skin tissues were fixed in 10% formaldehyde and stained with hematoxylin and eosin to confirm neutrophil infiltration (Kang et al., 2015). Congo red or toluidine blue were also used for staining eosinophils or mast cells in tissue, respectively (Kang et al., 2015). The stained tissues were examined using a Panoramic Viewer as described previously (3DHISTECH, Ltd., Budapest, Hungary) (Jang et al., 2019; Kim et al., 2019).

### mRNA Expression of Cytokines or Chemokines in Skin Samples

To measure the mRNA expression of various cytokines or chemokines in the skin, total RNA from homogenates of skin samples was extracted using an Easy-spin Total RNA Extraction Kit (Intron, Seoul, Korea) and reverse-transcribed to complementary DNA (cDNA) using a High-Capacity RNA-to-cDNA Kit (Thermo Fisher Scientific, Waltham, MA, USA) according to the manufacturer's instructions. Cytokine levels were measured using real-time polymerase chain reaction (real-time PCR) using a Rotor-Gene Q (Qiagen, Hilden, Germany) and a QuantiTect SYBR Green PCR kit (Qiagen) with 0.01 mM primers (Table S2) under the following conditions: initial denaturation at 95°C for 10 min, followed by 40 cycles of 95°C for 5 s and 60°C for 10 s. The relative expression levels of each cytokine or chemokine were calculated using the 2^−ΔΔCT^ method and normalized to the expression levels of hypoxanthine-guanine phosphoribosyltransferase (HPRT), as described previously (Livak and Schmittgen, 2001).

### Flow Cytometry Analysis in Treg Populations

Flow cytometry analyses were performed as described previously (Jang et al., 2019; Kim et al., 2019). Briefly, MLN tissues were smashed and filtered using a cell strainer (100 μm pore size (SPL Life Sciences Co., Ltd., Pocheon-si, Gyeonggi-do, Republic of Korea) and isolated T cells were subjected to a FC gamma receptor blocking and surface staining for 30 min at 4°C. To identify the live cells, T cells were stained with Fixable Viability Stain 510 (FVS510; BD Biosciences, San Jose, CA, USA) following the manufacturer's instructions. Moreover, T cell surface were stained with CD3+ fluorescein isothiocyanate (145-2C11; BD Biosciences), CD4+ Percep-Cyanine 5.5 (RM4-5; BD Biosciences) and CD25+ phycoerythrin (PC61; BD Biosciences) following the manufacturer's instructions. T cells were permeabilized with fixation/permeabilization buffer (eBioscience, San Diego, CA, USA) for intracellular staining and were stained with Foxp3+ Alexa Flour 647 (MF23; BD Biosciences) following the manufacturer's instructions. IgG isotypes (BD Biosciences) were used as a control. The CD4+CD25+Foxp3+ T cell population was counted using a BD FACSVerse™ Flow Cytometer (BD Biosciences).

### Cecum Microbiota Analysis

Cecum microbiota were analyzed as previously described with minor modifications (Lim et al., 2016; Jang et al., 2019; Kim et al., 2019). Briefly, total DNA was collected using a QIAmp Fast DNA stool mini kit (Qiagen) following the manufacturer's protocol. The V3-4 hypervariable region of the 16S rRNA gene was amplified using the barcoded primers 341F and 805R as described previously with minor modifications. The amplicons were purified subsequently using a QIAquick PCR Purification Kit (Qiagen) and sequenced using a MiSeq platform (Illumina. Inc., San Diego, CA, USA). Data were analyzed by using Quantitative Insights into Microbial Ecology 1.8.0 (QIIME) software (QIIME development team; http://qiime.org/)(Caporaso et al., 2010) and Greengenes version 13_5 database (http://greengenes.secondgemone.com). First, the sequences were clustered using an open-reference operational taxonomic unit (OTU) picking protocol at least 97% nucleotide identity with exclusions of singletons and rare OTUs, which were founded in <10% of samples. Then, the relative abundances of microbial taxa in samples were calculated from the non-rarefied OTU table. Alpha diversity was confirmed using the Chao1 index. Beta diversity was suggested based on the UniFrac distance between samples and visualized based on the weighted principle coordinate analysis (PCoA). Diversities were estimated from a rarefied OTU table, grouped into the mice with different treatments. Different taxa with significance were measured using linear discriminant analysis effect size analysis (LEfSe) (threshold >3.0) with Galaxy ver. 2.1.1 (Hutlab; http://huttenhower.org/galaxy) (Segata et al., 2011) and comparisons of abundances for significantly different microbial taxa among experimental groups were also measured.

### Measurement of AAs or SCFAs in Cecum Samples

AAs in cecum samples were analyzed as previously described (Jang et al., 2019). Briefly, approximately 1 mL of cecum extracts (concentration: 20 mg/mL) in liquid chromatography-grade methanol were derivatized by 70 μL of AccQ•Tag™ Ultra Borate Buffer (Waters Corporation., Milford, MA, USA) and 20 μL of AccQ•Tag™ Ultra reagent (Waters Corporation) for 10 min at 55°C. Samples were subsequently analyzed using an Acquity ultra-performance liquid chromatography (UPLC) (Waters Corporation) and a SYNAPT G2-Si mass spectrometer (Waters Corporation) with an ESI probe under the following conditions: 1.5 kV of capillary voltage, 600 and 50 L/h of desolvation or cone gas flow, respectively, and 250°C of desolvation temperature. An AA-S-18 analytical standard mixture (Sigma-Aldrich, St. Louis, MO, USA) was used to identify AAs, and data analyses were performed using MassLynx software 4.1 (Waters Corporation) (Roucher et al., 2013).

To measure SCFAs in samples, ceca were homogenized in distilled water and centrifuged at 13,000 × g for 5 min. Supernatant was collected and internal standards (1% 2-methylpentanoic acid for volatile acids or benzoic acid for non-volatile acids, respectively) were added. Then, extraction solvents (ethyl ether for volatile acids or chloroform for non-volatile acids, respectively) were added to the solution and centrifuged at 13,000 × g for 5 min. The organic layer was collected and analyzed using an Agilent 7890A gas chromatograph (Agilent Technologies, Santa Clara, CA, USA) under the following conditions; 1.5 kV of capillary voltage. Six hundred and fifty liter per hour of desolvation or cone gas flow, respectively, 170°C of oven temperature and 225°C for a flame ionization detector (FID) and an injection port temperature. The standard mixture was used as a reference for retention times and peak areas for extracts (David et al., 2014).

### Statistical Analysis

Experimental data were expressed as means ± standard error of the mean (SEM) of experimental groups (nine mice per group). When appropriate, data were analyzed using the Mann-Whitney *U* test. *P*-values < 0.05 were considered as statistically significant. GraphPad PRISM 5 (GraphPad software, San Diego, CA) were used for statistical analyses and visualizations.

## Results

### Effects of *L. fermentum* KBL375 on Clinical Symptoms or Immune Cells Using the *in vivo* AD Model

**Figure 1:**
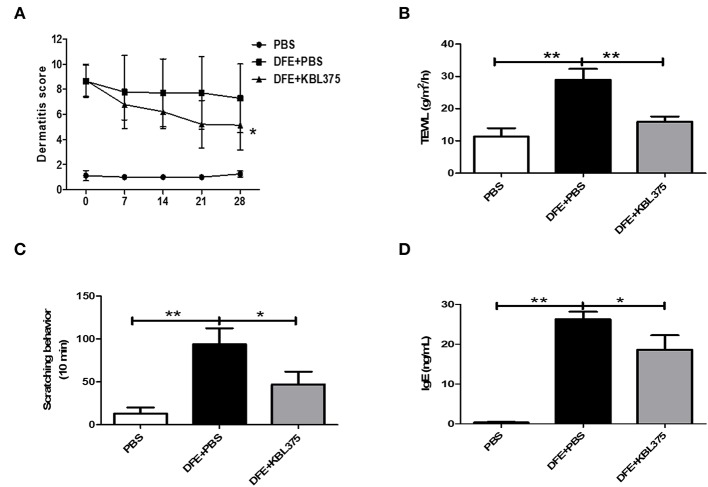
The effects of *Lactobacillus fermentum* (*L. fermentum*) KBL375 on clinical symptoms and immunoglobulin E (IgE) levels in an *in vivo* atopic dermatitis (AD) mouse model. **(A)** Dermatitis score; **(B)** Transepidermal water loss (TEWL); **(C)** Scratching behavior; **(D)** Serum IgE level. Firstly, we induced AD in male NC/Nga mice using 100 mg of *Dermatophagoides farinae* extract (DFE) cream twice a week for 21 days. Then, approximately 1 × 10^9^ colony forming units (CFU) of *L. fermentum* in 200 μL of PBS was administered to mice via oral gavage daily for 28 days, with weekly application of DFE cream. Dermatitis scores for mice were measured once a week. Prior to sacrifice, the TEWL scores and scratching behaviors in 10 min were measured. After 28 days of *L. fermentum* treatment, the serum IgE levels were measured using an enzyme-linked immunosorbent assay (ELISA). Data were expressed as the means ± SEM of experimental groups (nine mice per group). Asterisks indicate a statistical significance (^*^P < 0.05; ^**^P < 0.01; Mann-Whitney *U* test compared to mice treated with DFE + PBS as a positive control).

**Figure 2:**
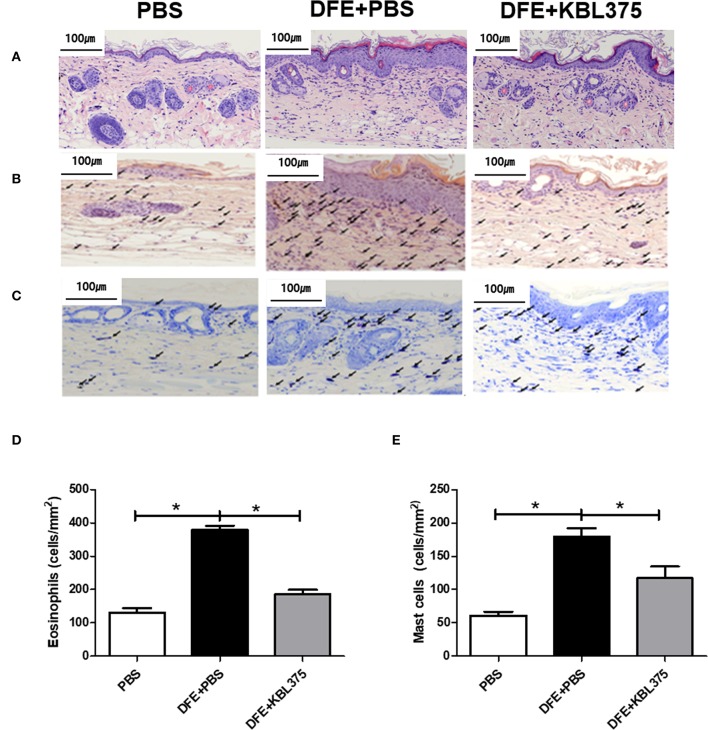
Improvements in dorsal skin inflammation via *L. fermentum* KBL375 treatment in the *in vivo* AD model. **(A)** Hematoxylin and eosin staining; **(B)** Congo red-staining; **(C)** Toluidine blue staining; **(D)** Eosinophils; **(E)** Mast cells. First, the skin samples were fixed using 10% formaldehyde and stained. Congo red or toluidine blue were used for staining eosinophils or mast cells, respectively. Black arrows indicate eosinophils or mast cells in skin samples. When appropriate, data were expressed as the means ± SEM of experimental groups (nine mice per group). Asterisks indicate a statistical significance (^*^P < 0.05; Mann-Whitney *U* test compared to mice treated with DFE + PBS as a positive control).

After 28 days of treatment, AD symptoms including hypertrophy and hyperkeratosis in the DFE + *L. fermentum* KBL375-treated mice were decreased with the reduction in ear or dorsal skin thickness compared to DFE + PBS-treated mice (*P* < 0.01 or *P* < 0.05, respectively) (Figure S1). Moreover, the DFE + *L. fermentum* KBL375-treated mice showed significant decreases in dermatitis score, with an average of six, compared to the DFE + PBS-treated group (*P* < 0.05) (Figure 1A). TEWL, scratching behaviors in 10 min, and serum IgE levels recovered after the *L. fermentum* KBL375 treatment (Figures 1B–D). Figure 2 also shows that the number of eosinophils and mast cells of dorsal skin tissues from DFE + *L. fermentum* KBL375-treated mice were significantly decreased compared to those in DFE + PBS-treated mice (*P* < 0.05).

### Effects of *L. fermentum* KBL375 on the Expression of Cytokines or Chemokines in Skin From the *in vivo* AD Model

**Figure 3:**
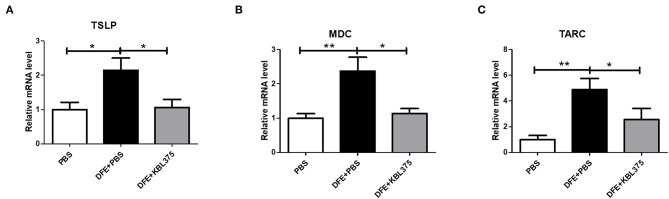
The effects of *L. fermentum* KBL375 on the expression of innate cytokines or chemokines in skin. **(A)** Thymic stromal lymphopoietin (TSLP); **(B)** Macrophage-derived chemokine (MDC); **(C)** Thymus and activation regulated chemokine (TARC). Briefly, total RNA from homogenates of skin samples was extracted and reverse-transcribed to complementary DNA (cDNA). Then, cytokine levels were measured using real-time polymerase chain reaction (real-time PCR) and normalized using hypoxanthine-guanine phosphoribosyltransferase (HPRT). Data were expressed as the means ± SEM of experimental groups (nine mice per group). Asterisks indicate a statistical significance (^*^P < 0.05; ^**^P < 0.01; Mann-Whitney *U* test compared to mice treated with DFE + PBS as a positive control).

**Figure 4:**
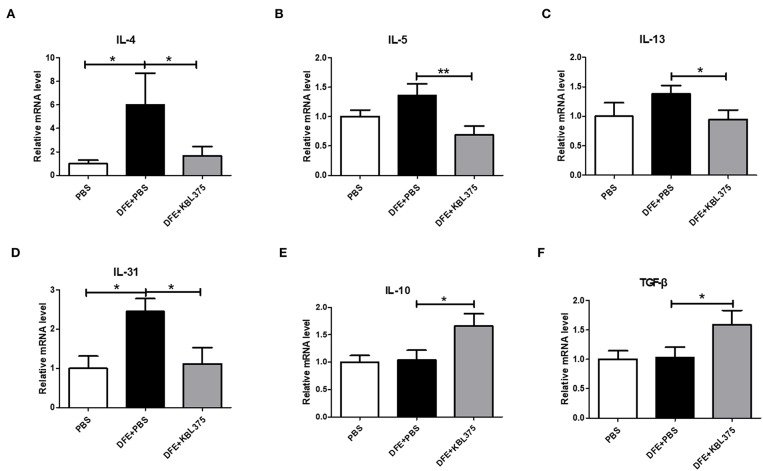
The effects of *L. fermentum* KBL375 on cytokine expression related to T helper (Th) 2 cells, IL-10, or transforming growth factor (TGF)-β. **(A)** Interleukin (IL)-4; **(B)** IL-5; **(C)** IL-13; **(D)** IL-31; **(E)** IL-10; **(F)** TGF-β. Cytokine levels were measured by real-time PCR and normalized using HPRT. Data were expressed as the means ± SEM of experimental groups (nine mice per group). Asterisks indicate a statistical significance (^*^P < 0.05; ^**^P < 0.01; Mann-Whitney *U* test compared to mice treated with DFE + PBS as a positive control).

Figure 3 shows the effects of *L. fermentum* KBL375 treatment on the expression of innate cytokine TSLP or chemokines including MDC or TARC in skin in the *in vivo* AD model. Skin samples from AD-induced mice treated with *L. fermentum* KBL375 showed significant reductions in mRNA levels of TSLP, MDC, or TARC compared to controls. Moreover, *L. fermentum* KBL375 showed strong down-regulatory effects on Th2-related cytokines including IL-4, IL-5, IL-13 and IL-31, and significant up-regulatory effects on anti-inflammatory cytokine IL-10 in AD-induced mice (*P* < 0.05) (Figure 4). Treatment with *L. fermentum* KBL375 also resulted in significant down-regulation of Th1-or Th17-related cytokines including TNF, IL-6, interferon (IFN)-γ, and IL-17A in AD-induced mice (*P* < 0.05) (Figure S2).

### Changes in Treg Populations in MLN Samples and Foxp3+ Expression of Skin Tissues After the *L. fermentum* KBL375 Treatment

**Figure 5:**
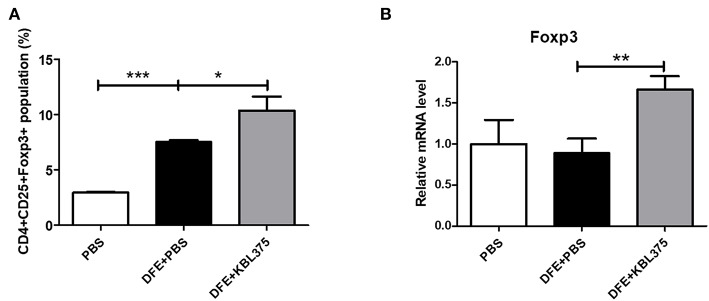
Flow cytometry analyses of T cells in mesenteric lymph nodes (MLNs) and Foxp3+ expression of skin tissues. **(A)** CD4+CD25+Foxp3+ T cell population; **(B)** Foxp3+ expression in skin samples in RNA level. T cells were collected in MLN tissues from sacrificed mice and initially stained using Fixable Viability Stain 510. Then, T cell surfaces were stained with CD3+ fluorescein isothiocyanate, CD4+ Percep-Cyanine5.5, and CD25+ phycoerythrin. Permeabilized T cells were also stained with Foxp3+ Alexa Fluor 647 for intracellular staining. The CD4+CD25+Foxp3+ T cell population was analyzed using a flow cytometer and the Foxp3+ mRNA levels were measured using real-time PCR and normalized using HPRT. Data were expressed as the means ± SEM of experimental groups (9 mice per each group). Asterisks indicate a statistical significance (^*^P < 0.05; ^**^P < 0.01; ^***^P < 0.001; Mann-Whitney *U* test compared to mice treated with DFE + PBS as a positive control).

Figure 5A shows the CD4+CD25+Foxp3+ Treg populations in MLN samples from AD-induced mice. Compared to DFE + PBS-treated mice, CD4+CD25+Foxp3+ Treg populations in MLN were significantly increased in *L. fermentum* KBL375 treated mice (*P* < 0.05). Moreover, Foxp3+ expression in skin tissues also significantly increased in DFE + *L. fermentum* KBL375-treated mice compared to DFE + PBS-treated mice (*P* < 0.05) (Figure 5B).

### Changes in Cecum Microbiota in the *in vivo* AD Model After the *L. fermentum* KBL375 Treatment

**Figure 6:**
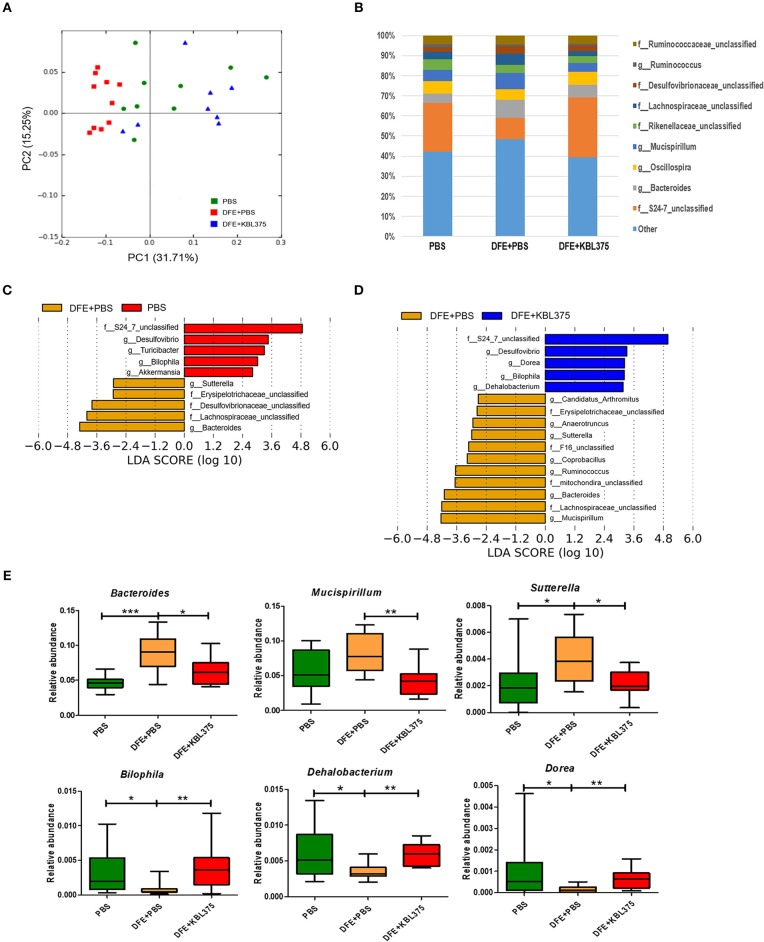
The effects of *L. fermentum* KBL375 on cecum microbiota of mice. **(A)** Principal coordinate analysis (PCoA) plot of the cecal microbiota with weighted UniFrac distance; **(B)** Taxonomic shifts in cecum microbiota for *L. fermentum* KBL375 treatment at the genus level; **(C)** Different taxa with significance measured using linear discriminant analysis effect size analysis for mice treated with PBS compared to mice treated with DFE + PBS (threshold >2.5); **(D)** Different taxa with significance measured using linear discriminant analysis effect size analysis for mice treated with DFE + *L. fermentum* KBL375 compared to mice treated with DFE + PBS (threshold >2.5); **(E)** Comparison of abundances of significantly different microbial taxa. Asterisks indicate a statistical significance (^*^P < 0.05; ^**^P < 0.01; ^***^P < 0.001; Mann-Whitney *U* test compared to mice treated with DFE + PBS as a positive control).

Figure 6 shows changes in bacterial communities in the cecum from AD-induced mice. PCoA plots showed that the cecum microbiota from DFE + *L. fermentum* KBL375-treated mice were similar to those from PBS-treated mice, which were clearly distinguishable from those of with DFE + PBS-treated mice (Figure 6A). S24-7 family was the dominant bacterial group in PBS-treated or DFE + *L. fermentum* KBL375-treated mice (24.4 and 29.8%, respectively) (Figure 6B). However, in DFE + PBS-treated mice, the abundance of S24-7 family was clearly decreased (10.7%), and a high proportion of genus *Bacteroides* was observed (8.9%) (Figure 6B). The cecum samples from PBS-treated and DFE + *L. fermentum* KBL375-treated mice showed significantly higher relative abundance of genus *Bilophila, Dehalobacterium*, and *Dorea* than DFE + PBS-treated samples (Figures 6C–E). On the other hand, cecum microbiota from DFE + PBS-treated mice showed a higher proportion of genus *Bacteroides, Mucisprillum*, and *Sutterella* compared to the other experimental groups (Figures 6C–E).

### Changes in Metabolic Pathways Predicted Using Microbial Compositions or Metabolites in the Cecum of the *in vivo* AD Model After the *L. fermentum* KBL 375 Treatment

**Figure 7:**
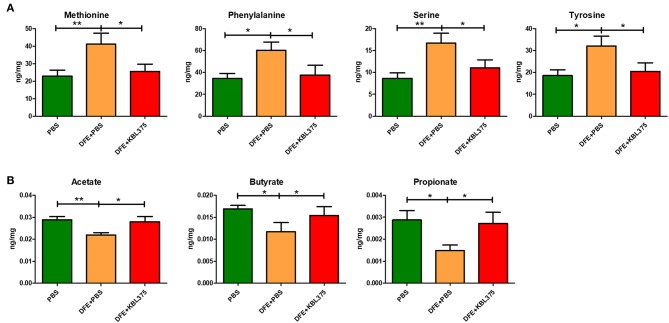
The effects of *L. fermentum* KBL375 on functional alterations related to various metabolites. **(A)** Amino acids; **(B)** Short-chain fatty acids. Amino acids or short-chain fatty acids in cecum samples were measured appropriately using an Acquity ultra performance liquid chromatograph with a SYNAPT G2-Si mass spectrometer or an Agilent 7890A gas chromatograph, respectively. Asterisks indicate a statistical significance (^*^P < 0.05; ^**^P < 0.01; Mann-Whitney *U* test compared to mice treated with DFE + PBS as a positive control).

Figure 7 shows the predicted changes in metabolic pathways after the *L. fermentum* KBL375 treatment in the ceca of AD-induced mice. The concentrations of various AAs, including methionine, phenylalanine, serine, and tyrosine, were significantly lower in ceca from AD-induced mice after *L. fermentum* KBL375 treatment in ceca from DSS + PBS-treated mice (Figure 7A). Significantly higher concentrations of three SCFAs, including acetate, butyrate and propionate were also measured in the ceca with the *L. fermentum* KBL375 treatment, similar to the results in PBS-treated mice (Figure 7B).

## Discussion

This study clearly demonstrated that the *L. fermentum* KBL375 treatment showed various protective effects against AD, such as improvements of clinical symptoms, immunomodulation of the host, and changes in metabolic pathways due to gut microbiota restoration. The DFE-induced AD mice showed severe physiological dysfunction, including skin barrier disruption, thickness, and dryness as previously suggested (Figure 1 and Figure S1) (Saeki et al., 2009). However, after 28 days of *L. fermentum* KBL375 administration, clinical features including dermatitis score, TEWL, scratching behavior, and serum IgE levels, which are considered typical abnormalities induced by AD, improved significantly (Figure 1) (Brandt, 2011; Lee and Yu, 2011; Zheng et al., 2011; Galli and Tsai, 2013). Previous studies reported that IgE-mediated activation of mast cells subsequently led to infiltration of eosinophils into skin lesions, which can contribute to the development of clinical features of AD (Matsuda et al., 1997; Galli and Tsai, 2013). Our results indicated that treatment of *L. fermentum* KBL375 inhibited the infiltration of eosinophils and mast cells (Figure 2).

Moreover, the DFE + *L. fermentum* KBL375-treated mice showed reductions in innate cytokine TSLP or chemokine MDC or TARC levels in skin (Figure 3). TSLP is known to be an activation factor for DCs, and activated DCs can boost the production of Th2-attracting chemokines including TARC and MDC, which can mediate Th2 recruitment into skin lesions (Pineiro and Stanton, 2007; Olszak et al., 2012; Tatsuno et al., 2015). Our results showed that *L. fermentum* KBL375 treatment can decrease the expression of not only Th2-related cytokines, including IL-4, IL-5, IL-13 and IL-31 but also other cytokines related to pro-inflammation, such as Th1- or Th17 (Figure 4 and Figure S2). Pro-inflammatory cytokine TNF and IL-6 contribute to inflammatory responses due to AD (Chang et al., 2013; Kim et al., 2013) and the responses of Th-related cytokines are important in the pathogenesis of AD (Grewe et al., 1998; Takaoka et al., 2006; Peng and Novak, 2015). On the other hand, *L. fermentum* KBL375 treatment showed the up-regulation of anti-inflammatory cytokine IL-10 and TGF-β in AD-induced mice indicating that the suppression of immune responses were occurred (Figure 4) (Agrawal et al., 2011; Kosaka et al., 2011; Purchiaroni et al., 2013). Therefore, *L. fermentum* KBL375-induced modulations in various cytokines or chemokines could be one of the major protective effects against AD.

Additionally, *L. fermentum* KBL375 treatment significantly increased CD4+CD25+Foxp3+ Treg populations in MLNs and skin tissues (Figure 5). As an effective suppressor of inflammation, CD4+Foxp3+ Treg populations are migrate and increase anti-inflammatory cytokines (Kim and Ji, 2012). Previous studies have also reported that probiotics can induce the generation of tolerogenic DCs, which are highly associated with IL-10 production and Treg differentiation (Kwon et al., 2010). Therefore, oral administration of *L. fermentum* KBL375 can induce strong protective effects for AD through immunomodulation.

Previous studies have shown that the interaction between a host and their microbiota is important to maintain the healthy status of the host (Sommer and Bäckhed, 2013). In this study, we investigated the changes in bacterial abundance and diversities in AD-induced mice after *L. fermentum* KBL375 treatment and confirmed that DFE + *L. fermentum* KBL375-treated mice had similar microbiota to PBS-treated mice, indicated that the restoration or protection of cecum microbiota were occurred by the *L. fermentum* KBL375 treatments in AD-induced mice (Figures 6A,B). Especially, S24-7 family, showed a clear reduction in a DFE + PBS-treated mice, but was dominant in ceca from both DFE + *L. fermentum* KBL375-treated and PBS-treated mice (Figure 6B). High relative abundances of genus *Bilophila*, which showed a negative correlation with lipopolysaccharide [LPS]- or *Candida albicans*-induced TNF-α production (Biancheri and Watson, 2017), and *Dehalobacterium*, which is associated with a protective effect against atherosclerosis (Chan et al., 2016), were observed in DFE + *L. fermentum* KBL375-treated mice (Figures 6C–E). Moreover, the genus *Bacteroides, Mucispirillum*, and *Sutterella* were significantly more abundant in DFE + PBS-treated mice than in DFE + *L. fermentum* KBL375-treated mice (Figures 6C–E). The abundance of genus *Bacteroides* is highly correlated with the occurrence of colitis (Lucke et al., 2006). Previous studies have also reported that genus *Mucispirillum* showed the an association with chemical-induced or *Citrobacter rodentium*-induced colitis in mice (Loy et al., 2017) and *Sutterella* species were associated with low secretory IgA levels due to their ability to degrade of both IgA and IgA-associated peptides (Levy et al., 2017). Taken together, these results indicate that *L. fermentum* KBL375 treatment could have positive effects in cecum microbiota altered by AD.

Our results elucidated that *L. fermentum* KBL375 treatment has shown the increases of metabolites such as AAs and SCFAs (Figure 7). Microbial metabolites such as AAs from intestinal microbiota play important roles in host physiology and immunity (Dai, 2011; Rooks and Garrett, 2016). Moreover, AAs, which are produced by instestinal microbiota, can serve as precursors for SCFA synthesis (Neis et al., 2015). SCFAs are used as energy sources, substrates or signal molecules, and are strongly associated with the metabolism of lipids, glucose, and cholesterols (Kim et al., 2017; McNabney and Henagan, 2017). Especially, butyrate, an important SCFA that modulates immune responses in macrophages or promotes the formation of Tregs, can be produced by family *Lachnospiraceae* or *Ruminococcaceae* including genus *Dorea* or *Oscillospira*, respectively (Cushing et al., 2015; Blacher et al., 2017; Kang et al., 2017). Therefore, *L. fermentum* KBL375 treatment can induce the increases of AAs, including methionine, phenylalanine, serine, and tyrosine, and SCFAs including acetate, butyrate, and propionate, in the intestines of AD-induced mice. However, further longitudinal studies with a large number of animal or human subjects and various study designs should be performed to elucidate the effects of *L. fermentum* KBL375 on microbiota or microbiota-related metabolites.

In conclusion, *L. fermentum* KBL375 treatment showed strong anti-AD effects in the *in vivo* mouse model via modulation of the immune system and induction of various metabolites. Therefore, *L. fermentum* KBL375 could be a useful to ameliorate AD through with various applications such as functional foods or drugs.

## Data Availability Statement

The datasets for this study can be found in the: https://www.ncbi.nlm.nih.gov/bioproject/558163.

## Ethics Statement

All experimental procedures for AD-induced *in vivo* mouse models were approved by the Institutional Animal Care and Use Committee (IACUC: SNU-160928-1-1) of Seoul National University, Republic of Korea.

## Author Contributions

W-KK, YJ, and GK mainly designed the study, and other authors contributed to data acquisition and manuscript preparation. GK supervised this study.

### Conflict of Interest

GK was the founder of KoBioLabs, Inc., and SP was employed by KoBioLabs, Inc. The remaining authors declare that the research was conducted in the absence of any commercial or financial relationships that could be construed as a potential conflict of interest. The reviewer TU declared a past co-authorship with one of the authors DH to the handling editor.
