# Supplementary material for: Administration of Lactobacillus fermentum KBL375 Causes Taxonomic and Functional Changes in Gut Microbiota Leading to Improvement of Atopic Dermatitis
Source: Front Mol Biosci. 2019 Sep 27;6:92. doi: 10.3389/fmolb.2019.00092 (PMC6777006; doi:10.3389/fmolb.2019.00092)
Supplement: Supplementary file 1 [file Table_1.DOCX]

**Supplementary Table 1.** Calculated dermatitis score (Maximum score = 12).

| **Content** | **Score** | **Clinical severity** |
| --- | --- | --- |
| **Erythema/Hemorrhage** | 0 | None |
|  | 1 | Mild |
|  | 2 | Moderate |
|  | 3 | Severe |
| **Scaling/Dryness** | 0 | None |
|  | 1 | Mild |
|  | 2 | Moderate |
|  | 3 | Severe |
| **Edema** | 0 | None |
|  | 1 | Mild |
|  | 2 | Moderate |
|  | 3 | Severe |
| **Excoriation/Erosion** | 0 | None |
|  | 1 | Mild |
|  | 2 | Moderate |
|  | 3 | Severe |

**Supplementary Table 2.** Primers used in this study.

| **Target** | **Sequence** | **Reference** |
| --- | --- | --- |
| IL-6 | Fw^a^: 5’-CTG CAA GAG ACT TCC ATC CAG-3’ | (Chang et al., 2013) |
|  | Rv^b^: 5’-AGT GGT ATA GAC AGG TCT GTT GG-3’ |  |
| HPRT | Fw: 5′-TTA TGG ACA GGA CTG AAA GAC-3′ | (Kwon et al., 2010) |
|  | Rv: 5′-GCT TTA ATG TAA TCC AGC AGG T-3′ |  |
| Foxp3 | Fw: 5’-CCC ATC CCC AGG AGT CTT G-3’ |  |
|  | Rv: 5’-CCA TGA CTA GGG GCA CTG TA-3’ |  |
| IFN-γ | Fw: 5′-TCA AGT GGC ATA GAT GTG GAA GAA-3′ |  |
|  | Rv: 5′-TGG CTC TGC AGG ATT TTC ATG-3′ |  |
| IL-4 | Fw: 5’-ACA GGA GAA GGG ACG CCA-3’ |  |
|  | Rv: 5’-GAA GCC CTA CAG ACG AGC TCA-3’ |  |
| IL-5 | Fw: 5′-TCC AAT GCA TAG CTG GTG ATT T3′ |  |
|  | Rv: 5′-AGC ACA GTG GTG AAA GAG AC-3′ |  |
| IL-10 | Fw: 5′-TCA TTT CCG ATA AGG CTT GG-3′ |  |
|  | Rv: 5′-ATA ACT GCA CCC ACT TCC CA-3′ |  |
| IL-13 | Fw: 5’-GCA ACA TCA CAC AGG ACC AGA-3’ |  |
|  | Rv: 5’-GTC AGG GAA TCC AGG GCT AC-3’ |  |
| IL-17A | Fw: 5′-TTC ATC TGT GTC TCT GAT GCT-3′ |  |
|  | Rv: 5′-TTG ACC TTC ACA TTC TGG AG-3′ |  |
| TGF-β | Fw: 5′-GAA GGC AGA GTT CAG GGT CTT-3′ |  |
|  | Rv: 5′-GGT TCC TGT CTT TGT GGT GAA-3′ |  |
| TNF-α | Fw: 5′-CAT CTT CTC AAA ATT CGA GTG ACA A-3′ |  |
|  | Rv: 5′-TGG GAG TAG ACA AGG TAC AAC CC-3′ |  |
| MDC | Fw: 5'-CCA AGG TGC CTT TGA AGA CT-3' | (Olszak et al., 2012) |
|  | Rv: 5'-TCC TCC AGC TGG TGG TTA CT-3' |  |
| TARC | Fw: 5'-CAG GAA GTT GGT GAG CTG GTA TA-3' | (Pineiro et al., 2007) |
|  | Rv: 5'-TTG TGT TCG CCT GTA GTG CAT A-3' |  |
| IL-31 | Fw: 5′-ATA CAG CTG CCG TGT TTC AG-3′ | (Takaoka et al., 2006) |
|  | Rv: 5′- AGC CAT CTT ATC ACC CAA GAA-3′ |  |
| TSLP | Fw: 5'-CGA GCA AAT CGA GGA CTG TGA G -3' | (Xhao et al., 2012) |
|  | Rv: 5'-GCA GTC GTC ATT GAG CGC TTC-3' |  |

^a^Fw represents sequences of a forward primer

^b^Rv represents sequences of a reverse primer


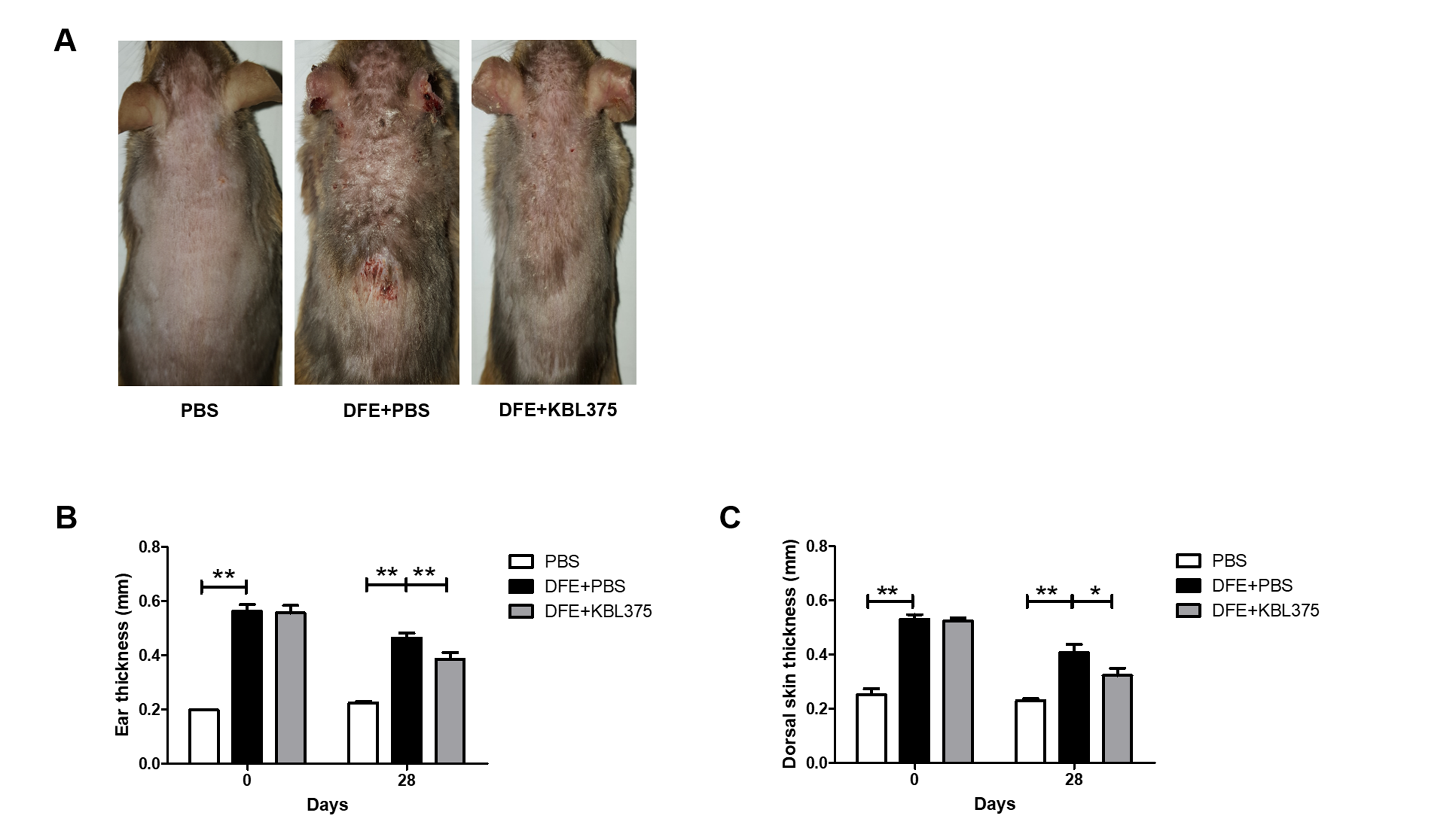


**Figure S1.** Improvements of clinical symptoms in the *in vivo* atopic dermatitis (AD) mouse model via *L. fermentum* KBL375 treatment. (A) Photographs of mouse skin before sacrifice; (B) Changes in ear thickness; (C) Changes in dorsal skin thickness. Data were expressed as the means ± SEM of experimental groups (nine mice per group). Asterisks indicate a statistical significance (*, *P* < 0.05; **, *P* < 0.01; Mann-Whitney *U* test compared to mice treated with DFE + PBS as a positive control).


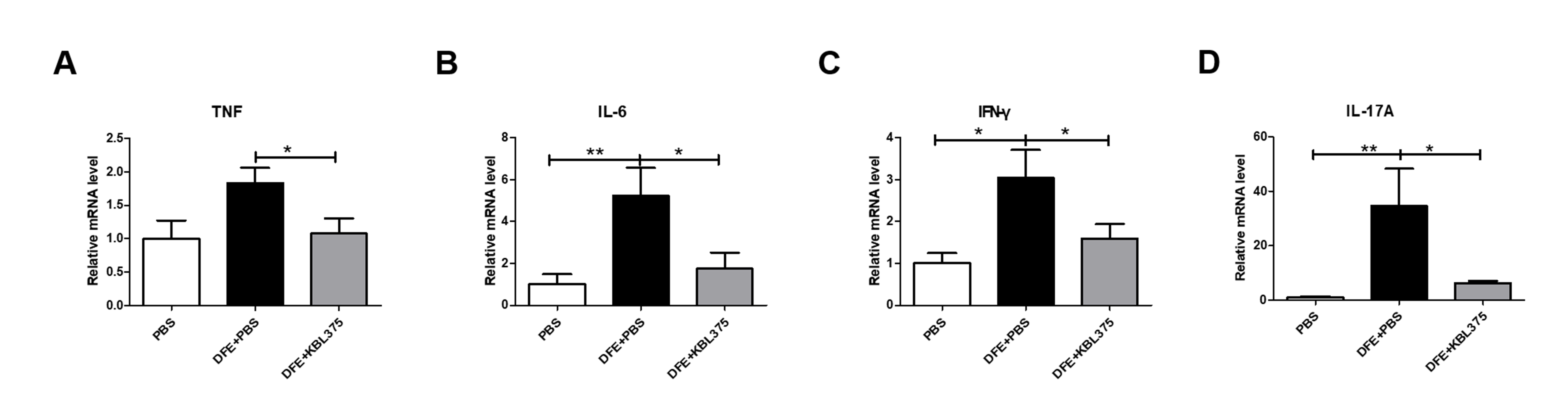


**Figure S2.** Changes in pro-inflammatory, T helper (Th) 1 or Th17 cell-related cytokines via *L. fermentum* KBL375 treatment. (A) Tumor necrosis factor (TNF); (B) Interleukin (IL)-6; (C) Interferon (IFN)-γ; (D) IL-17A. Cytokine levels were measured using reverse transcription polymerase chain reaction (PCR) and normalized using hypoxanthine-guanine phosphoribosyltransferase (HPRT). Data were expressed as the means ± SEM of experimental groups (nine mice per group). Asterisks indicate a statistical significance (*, *P* < 0.05; **, *P* < 0.01; Mann-Whitney *U* test compared to mice treated with DFE + PBS as a positive control).
